# Selective blockade of TGFbeta in the mice model of collagen-induced arthritis

**DOI:** 10.1186/1479-5876-9-S2-P34

**Published:** 2011-11-23

**Authors:** Elena Gonzalo, Gabriel Criado, Begoña Santiago, Jose Luis Pablos, Maria Galindo

**Affiliations:** 1 Laboratorio de Reumatología, Instituto de Investigación, Hospital 12 de Octubre, Madrid, Spain

## Introduction

TGFβ contributes to Th17 and Treg development and has pro and anti-inflammatory properties. However, its role in rheumatoid arthritis is controversial. p17 is a short synthetic peptide that selectively inhibits TGFβ.

## Aim

To analyse the effect of TGFβ blockade with p17 in the murine model of collagen-induced arthritis (CIA), during induction of arthritis or clinical progression of the disease.

## Methods

Male DBA/1 mice were immunized intradermally with type-II collagen (CII) in Complete Freund's Adjuvant. To analyse the contribution of TGFβ to the induction of CIA, mice were treated daily with p17 or vehicle (PBS) during 3 weeks starting at the time of immunization. The role of TGFβ in progression of established arthritis was assessed by treating daily for 10 days starting on the day of arthritis onset. Signs of arthritis were monitored according to clinical and inflammation scales. Histological damage was analysed by hematoxylin-eosin and safranin staining. T cell population frequency was analysed by flow cytometry in lymph node cell suspensions. mRNA levels were analysed by RT-qPCR. The response to CII was quantified with WST-1. IL6 and anti-CII-Ab were quantified by ELISA. Statistical differences between groups were compared using Student’s t-test or Mann-Whitney U as required.

## Results

In the induction phase, p17 reduced arthritis severity, number of affected joints, histological joint inflammation and cartilage damage, but differences were not statistically significant (area under curve of clinical score change: PBS=48.24±8.34, p17=30.57±6.35; p=0,09). Blockade of TGFβ did not modify the frequency of Th1, Th2 and Th17 cells, but significantly reduced the percentage of Treg cells (PBS=7.79±0.17, p17=6.39±0.57; p=0,03). Levels of IL6, IL17, IFNγ and Foxp3 mRNA were comparable in spleen and joints. There were no significant differences in specific proliferation to CII or the production of IL6 and anti-CII-Ab IgG1/IgG2a. In the phase of established disease, p17 reduced the severity of arthritis although this difference was not statistically significant (PBS=29.04±5.28, p17=20.72±4.18; p=0,22). Finally, the blockade of TGFβ did not affect other parameters analyzed.

## Conclusion

Treatment of CIA with p17 does not have a statistically significant effect on the course of arthritis, suggesting that TGFβ plays a minor role in this pathology.

